# Comparison of the effectiveness of single- and multiple-sessions disinfection protocols against endotoxins in root canal infections: systematic review and meta-analysis

**DOI:** 10.1038/s41598-020-79300-3

**Published:** 2021-01-13

**Authors:** Gustavo G. Nascimento, Diego G. D. Rabello, Bruna J. M. Corazza, Ana P. M. Gomes, Eduardo G. Silva, Frederico C. Martinho

**Affiliations:** 1 Section of Periodontology, Department of Dentistry and Oral Health, Aarhus University, Aarhus, Denmark; 2 Endodontic Division, Department of Restorative Dentistry, Institute of Science and Technology, São Paulo State University, São José dos Campos, São Paulo Brazil; 3 Department of Social and Pediatric Dentistry, Institute of Science and Technology, São Paulo State University, São José dos Campos, São Paulo Brazil; 4 Endodontic Division, Department of Advanced Oral Sciences and Therapeutics, School of Dentistry, University of Maryland, Baltimore, MD USA

**Keywords:** Root canal treatment, Endodontic files

## Abstract

This systematic review (SR) addressed the following common clinical question: What is more effective in reducing or eliminating endotoxin in endodontic infections—single or multiple-session treatments using calcium hydroxide medications? Literature searches of Medline/PubMed, Embase, Cochrane Library, Scielo, Science Direct, Web of Knowledge, Scopus, and Google Scholar databases. Two reviewers independently assessed the eligibility for inclusion, extracted data, and evaluated the quality of the studies using the risk of bias tools. Electronic searches resulted in 358 articles, of which 32 studies were included for full-text assessment, and nine were included in this review. Meta-analysis pooling all the nine studies revealed lower levels of endotoxin for multiple-session treatment (*P* < 0.001). The sub-group analysis indicated no difference between single-session and 7 days of Ca(OH)_2_ medication (SMD − 0.32; *P* = 0.22). However, 14-days (I^2^ = 80.5%, *P* < 0.001) and 30-days (I^2^ = 78.9%, *P* < 0.01) of Ca(OH)_2_ medication was more effective than single-session treatment (both, *p* < 0.001). Overall, Overall, this SR provides evidence to support that multiple-session disinfection protocols with the placement of Ca(OH)_2_ medications are more effective in reducing the levels of endotoxin from root canal infections compared to single-session when applied for 14 and 30 days.

## Introduction

Apical periodontitis is an inflammatory process of the periapical tissues caused mainly by bacteria and their byproducts in the root canal that can culminate in bone loss^1–5^. Studies have demonstrated that these infections are polymicrobial comprised both by Gram-positive and -negative bacteria^1–6^.

Lipopolysaccharides (LPSs), also known as endotoxins, are the major surface molecule and pathogenic factor of Gram-negative bacteria^7,8^. LPSs are present inside a bacterial cell attached to the outlying cell membrane. Endotoxins have been detected in 100% of teeth with root canal infections and the presence of apical periodontitis^9–12^. LPS is one of the most important virulent factors involved in the development of periapical inflammation^13,14^. Endotoxin activates immunocompetent cells present in periapical tissues leading to the release of several proinflammatory mediators^14^.

Higher levels of endotoxins present in root canal infections are strongly correlated to the presence of spontaneous pain^9,15^, clinical symptomatology such as pain on palpation and tenderness to percussion^15,16^, presence of exudation^9,15,16^, and severity of bone resorption (size/volume of periapical radiolucency)^11,16^.

Taken the high inflammatory potential of endotoxins to periapical tissues and their strong correlation with the presence of clinical symptoms as well as the severity of bone resorption, the removal/ elimination of endotoxins during root canal therapy is important for the remission of symptoms and healing of periapical tissues. Over the years, different studies have evaluated the ability of root canal therapies in reducing/eliminating endotoxins from infected root canals^10,12,17–21^. Single-session treatment accomplished by disinfection protocols involving chemomechanical preparation followed by prompt obturation and multiple-session therapy with the supplement of interappointment intracanal medication has been tested against endotoxins^12,22^.

Calcium hydroxide [Ca(OH)_2_] is the most commonly used intracanal medication for multiple-session therapy^12,17,18,20,23–25^. Its efficacy against endotoxins has been demonstrated by in vitro^23,26^ and animal studies^24,27^. Ca(OH)_2_ is able to hydrolyze lipid A molecule, an Esther-linked hydroxyl fatty acids, resulting in atoxic components^19,23,24,26^. A histopathological study has further corroborated this ability to convert lipid A into atoxic components. However, controversy exists on whether Ca(OH)_2_ intracanal medication can clinically boost the reduction or elimination of endotoxins from infected root canals achieved with root canal instrumentation^10,15,18,20–22,25,28–31^.

The assessment of the effectiveness of different root canal therapies on the reduction or elimination of endotoxins from root canal infections through this systematic review (SR) and meta-analysis is important to provide a high quality of evidence and relevant data to contribute to the establishment of evidence-based treatment protocols. Therefore, this SR aimed to compare the effectiveness of multiple-session treatment with the use of calcium hydroxide intracanal medications over single-session treatment against endotoxins present in root canal infections.

## Material and methods

This SR followed the Preferred Reporting Items for Systematic Reviews and Meta-Analyses (PRISMA) guidelines^32^ and registered in the PROSPERO database (Registration number: CRD42017077160).

### Research question

What is more effective in reducing or eliminating endotoxin in endodontic infections—single or multiple-session treatments using calcium hydroxide medications? This review question was based on the PICO format as follows: Population—patients with endodontic infection; Intervention—endodontic treatment; Comparison—multiple-session using calcium hydroxide medication versus single-session; Outcome—level of endotoxin.

### Search strategy

The literature search was conducted to identify clinical studies that compared the effectiveness of single- and multiple-session root canal therapy in reducing endotoxin from root canal infections. Electronic searches were conducted in Medline/PubMed, Embase, Cochrane Library, Scielo, Science Direct, Web of Knowledge, Scopus databases, and Google Scholar. The literature search was performed retrieving articles published until Aug 2020. In addition to the electronic search, the reviewers undertook a hand search in the references list of each included study. Search was conducted with the following MeSH and free terms: (“periapical periodontitis”[all], OR “periapical diseases”[all], OR “dental pulp diseases”[all], OR “apical periodontitis”[all], OR “endodontic infection”[all]) AND (“endotoxin”[all], OR “LPS”[all], OR “lipopolysaccharide”[all]) AND (“intracanal medication”[all], OR “calcium hydroxide”[all]).

### Eligibility criteria

Clinical studies in humans that compared the effect of single-session versus multiple-session using Ca(OH)_2_ intracanal medications were included in this SR. Studies were eliminated if they presented any of the following exclusion criteria: in vitro or animal study, case report, review or opinion article; absence of the endotoxin levels; absence of the description of the type of endodontic infection; lack of a clear definition of the method used for endotoxin detection; absence of the amount of endotoxin in the same study before and after intracanal medication.

### Study selection procedure

All references were tabulated by using the software EndNote X7 (Thomson Reuters, New York, NY, US). Duplicate references were excluded. Titles, abstracts, and keywords were screened by two reviewers (DDR and BJC) based on the eligibility criteria. The Kappa coefficient was performed to determine the agreement between the reviewers (kappa = 0.90). The screened lists were compared, and in case of any disagreement, a consensus was reached through discussion or consultation of a third author (GGN). After initial screening of titles and abstracts, the full articles were evaluated by the same two reviewers (DDR and BJC).

### Data collection process

Data extraction was performed independently by two reviewers (DDR and BJC). In case of disagreements, these were solved by discussion or seeking the opinion of a third author (GGN).

### Data extraction

The following information was gathered from the included articles: *Publication details*: authors and year of publication; *Characteristics of the study*: sample size and study design; *Characteristics of the patients*: diagnosis, age range of the patients, and teeth treated; *Characteristics of the endodontic treatment*: type of instrumentation, irrigation solution, type of vehicle used for the Ca(OH)_2_ medication, dosage [ratio of Ca(OH)_2_, number of renewal sessions, period of time of application Ca(OH)_2_ intracanal medication, and method used to deliver the medication inside the canal; and *Characteristics of the endotoxin detection*: method and test used for the detection of endotoxin.

### Risk of bias in individual studies

Quality assessment was performed independently by two reviewers (DDR and BJC). The risk of bias in randomized clinical trials (RCTs) was performed using the Cochrane risk of bias tool (RoB 2.0)^33^. The RoB 2.0 tool assesses the risks of bias according to 6 domains as follows: randomization process, deviations from the intended interventions, missing outcome data, measurement of the outcomes, selection of reported result and, overall. The included studies were judged as low risk of bias, high risk of bias, or some concerns. The study was considered a low risk of general bias if all domains were classified as low risk. If the study raised any concerns in at least one domain, it was considered to have some general concerns. If the study were at ‘high risk’ in any domain or three or more of the same in some concerns, it would be considered a ‘high risk bias.’ The risk of bias classification in non-randomized clinical studies was performed using the Cochrane risk of bias tool (ROBINS-I)^34^, according to 7 domains as follow: confounding; selection of participants; classification of interventions; deviations from the intended interventions; missing outcome data; measurement of outcomes; and selection of the reported results. For each domain, the studies were classified as low, moderate, serious, critical, or no information available for risk of bias. The general bias assessment was determined by combining the level bias in each domain. When the study was judge as ‘unclear’ in their key domains, an attempt was made to contact the authors to obtain more information and enable definitive judgment of ‘low’ or ‘high’ risk.

### Analytical approach

Articles that fulfilled all the inclusion criteria were considered for quantitative synthesis by means of meta-analysis. First, a meta-analysis was performed to combine a broad range of studies that used calcium hydroxide intracanal medications for 7, 14, and 30 days compared to single-session protocols to obtain. Subsequently, a subgroup meta-analysis was performed to stratify the pooled results by the period of time application of Ca(OH)_2_ intracanal medication: 7, 14, or 30 days compared to single-session therapy. Pooled estimates were calculated using both the fixed- and the random-effects model, and in the presence of heterogeneity (I^2^ > 50%; Chi-square and *P* value < 0.1), the latter was preferred^35^. The analyses were conducted using the software Stata SE 16.0 (StataCorp., College Station, TX, USA).

## Results

### Summary of included studies

**Figure 1:**
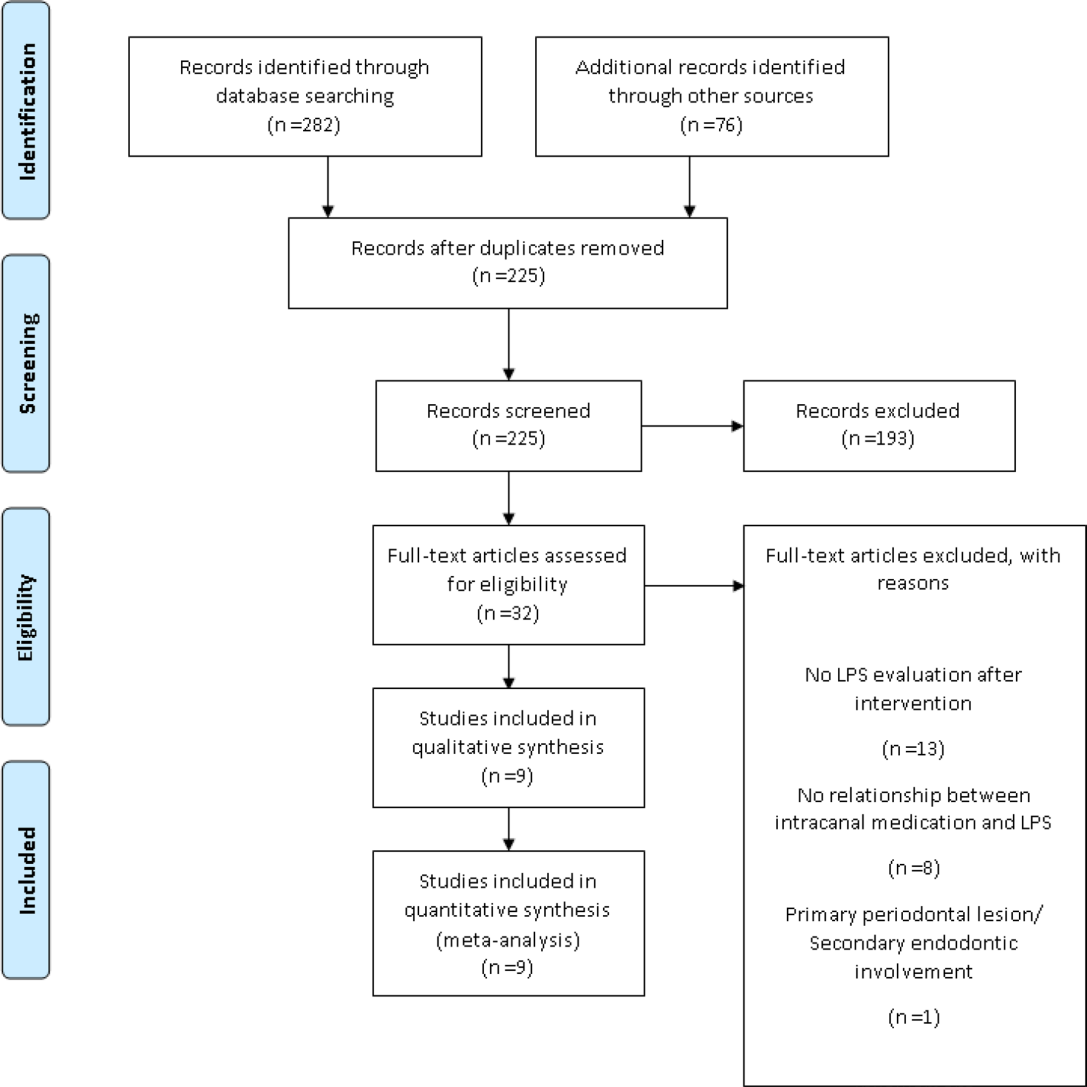
PRISMA flowchart of the study selection procedure.

The PRISMA flow chart summarizes the study selection process (Fig. 1). The initial electronic search revealed 358 articles. After excluding 133 duplicates, 225 articles remained for the title and abstract screening. Thirty-two studies were included for full-text assessment and, of those, 22 were excluded. Accordingly, nine studies fulfilled the inclusion criteria for this review. Table S1 displays the excluded studies and the main reason for exclusion.

### General characteristics

The nine studies included in this SR and meta-analysis^10,12,18,20–22,25,36,37^ consisted of a total of 426 patients, with 223 patients treated in single-session and 203 patients treated in multiple-session with Ca(OH)_2_ intracanal medications. All 9 clinical studies analyzed patients with primary endodontic infections with the presence of apical periodontitis. Out of the 9 studies, 5 were randomized clinical trials^10,12,22,25,37^ and four NRSis^18,20,21,36^. All studies used the Limulus Amebocyte Lysate (LAL) method for the quantification of endotoxin. The values of endotoxin were determined in Units of Endotoxin/mL (EU/mL).

Root canal instrumentation was performed using GT rotary files + k-files (Malleifer-Dentsply, Switzerland)^25^, Endo-Eze (Ultradent Products, USA) + k-files^18,21,22^, Mtwo (VDW, Germany)^10,36,37^, ProTaper (Dentsply, Switzerland)^20^, and Reciproc (VDW, Germany)^12^.

Root canal irrigation was performed using chlorhexidine (2% CHX-gel)^10,18,22,25,36,37^ or sodium hypochlorite (1% or 2.5% NaOCl)^10,12,20–22,37^. Final irrigation with 17%EDTA was performed in all studies except for Vianna 2007^25^ and Adl 2015^20^.

Calcium hydroxide intracanal medication was used for 7 days^20,25^, 14 days^12,18,21,22^ and 30 days^10,36,37^. The type of vehicle for the medication was either 2% CHX-gel^18,21,25,36^, saline solution^10,12,20,25,37^ or propylene glycol^22^. The medication dosage, the ratio of Ca(OH)_2_ to the vehicle, was 1:1^10,18,21,37^ or not described^12,20,22,25,36^. Additionally, the lentulo spiral was used to deliver the Ca(OH)_2_ medication in all 9 studies. The included studies are summarized in Table 1.

**Table 1:** Characteristics and main findings of the included studies.

Author/ year	Sample size	Study design	Diagnosis	Age range of the patients	17% EDTA Minutes	File system	Irrigant	Intracanal Medication	Intracanal Medication proportion	Method of delivery	Teeth treated	Method of endotoxin detection	Time of application of intracanal medication (days)
Vianna et al. (2007)	24 patients	RCT	Necrotic pulp with radiographic evidence of apical periodontitis	Ranged from 18 to 65 years	No	GT rotary files (Dentsply Sirona, USA) complemented by hand files	G1: 2% CHX gel	G1: Ca(OH)_2_ + SSL (n = 8)	Not described	Lentulo spiral	Single-rooted teeth	Endpoint Chromogenic LAL assay	7
							G2: 2% CHX gel (n = 8)						
							G3: Ca(OH)_2_ + 2% CHX gel (n = 8)						
Oliveira et al. (2012)	36 patients	NRSis	Pulp necrosis and radiographic apical periodontitis	Ranged from 19 to 55 years	Yes (3 min)	Endo-Eze system (Ultradent Products, USA), complemented by hand files	G1: 2% CHX gel + LW, (n = 12)	Ca(OH)_2_ + CHX gel	1:1	Lentulo spiral	Single-rooted teeth	Kinetic quantitative chromogenic LAL assay	14
							G2: 2% CHX gel + PmB, (n = 12)						
							G3: 2% CHX gel (n = 12)						
Xavier et al. (2013)	48 patients	RCT	Primary endodontic infections	Not described	Yes (3 min)	Endo-Eze system (Ultradent Products, USA), complemented by hand files	G1: 1% NaOCl (n = 24)	Ca(OH)_2_ + propylene glycol	Not described	Lentulo spiral	Single-rooted teeth	Kinetic quantitative chromogenic LAL assay	14
							G2: 2% CHX gel gel (n = 24)						
Sousa et al. (2014)	10 patients	NRSis	Infected root canals with acute apical abscesses	Not described	Yes (3 min)	Mtwo (VDW, Germany)	2% CHX gel	Ca(OH)_2_ + CHX gel	Not described	Lentulo spiral	Not described	Kinetic Turbidimetric LAL assay	30
Marinho et al. (2014)	30 patients	RCT	Pulp necrosis and radiographic evidence of apical periodontitis	Not described	Yes (3 min)	Mtwo (VDW, Germany	G1: 2.5% NaOCl (n = 10)	Ca(OH)_2_ + SSL	1:1	Lentulo spiral	Single-rooted teeth	Kinetic Turbidimetric LAL assay	30
							G2: 2% CHX gel (n = 10)						
							G3: SSL (n = 10)						
Adl et al. (2015)	24 patients	NRSis	Necrotic pulp with apical periodontitis	Ranged from 26 to 61 years	No	ProTaper (Dentsply Sirona, USA)	2.5% NaOCl	Ca(OH)_2_ + SSL	Not described	Lentulo spiral	Single-rooted teeth	Kinetic quantitative chromogenic LAL assay	7
Marinho et al. (2015)	30 patients	RCT	Pulp necrosis and radiographic evidence of apical periodontitis	Ranged from 18 to 55 years	Yes (3 min)	Mtwo (VDW, Germany)	G1: 2.5% NaOCl (n = 10)	Ca(OH)_2_ + saline solution	1:1	Lentulo spiral	Single-rooted teeth	Kinetic Turbidimetric LAL assay	30
							G2: 2% CHX gel (n = 10)						
							G3: Saline solution (n = 10)						
Carvalho et al. (2016)	33 patients	Clinical study	Radiographically visible apical periodontitis	Not described	Yes (3 min)	Endo-Eze system (Ultradent Products, USA), complemented by hand files	G1: 2.5% NaOCl + LW (n = 11)	Ca(OH)_2_ + 2% CHX gel	1:1	Lentulo spiral	Single-rooted teeth	Kinetic quantitative chromogenic LAL assay	14
							G2: 2.5% NaOCl + PmB (n = 11)						
							G3: 2.5% NaOCl (n = 11)						
Rabello et al. (2017)	24 patients	Randomised clinical trial	Pulp necrosis and radiographic evidence of apical periodontitis	Not described	Yes (3 min)	Reciproc (VDW, Germany)	2.5% NaOCl	Ca(OH)_2_ + saline solution	Not described	Lentulo spiral	Single-rooted teeth	Kinetic quantitative chromogenic LAL assay	14

### Risk of bias within studies

**Figure 2:**
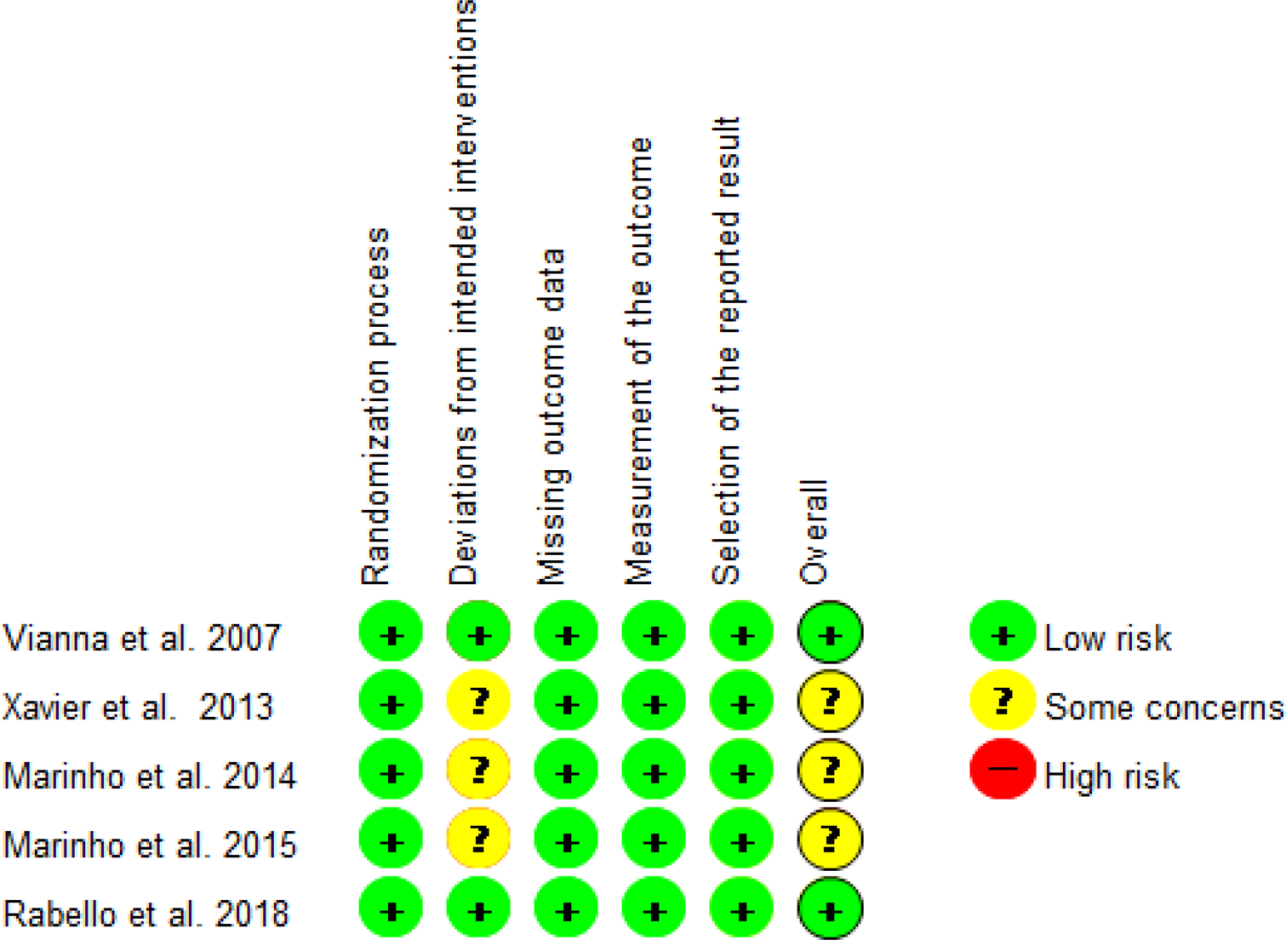
Bias risk assessment: summary of assessment according to the RoB2 tool.

**Figure 3:**
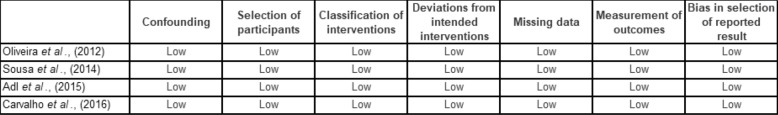
Bias risk assessment: summary of the assessment according to the ROBINS-I tool.

The assessment of the risk of bias of the selected randomized clinical trials (RCTs) was performed using the Cochrane risk of bias tool (RoB 2.0) assessing 6 different domains^33^ (Fig. 2). Of the 5 RCTs included, 3 of them were ‘overall’ classified as “some concerns” of bias and 2 “low risk” of bias. All 5 RCTs^10,12,22,25,37^ included in this SR were classified as ‘low risk’ of bias according to RoB 2.0. The assessment of the risk of bias of the non-randomized studies of interventions (NRSis)^18,20,21,36^ was performed according to ROBINS-I (Fig. 3). All NRSis included in this SR were classified as “low risk’ of bias.

### Quantitative synthesis

**Figure 4:**
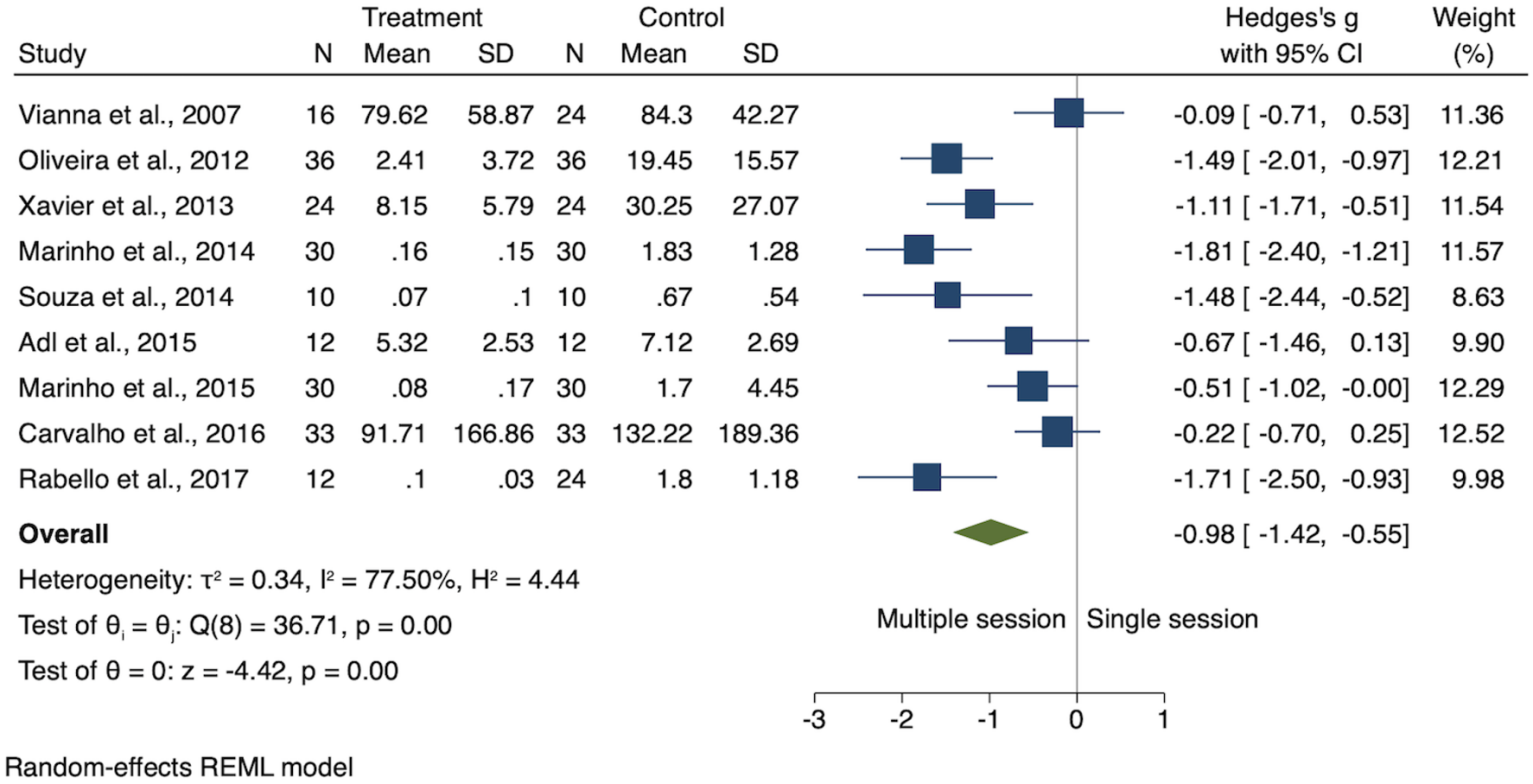
Forest plot for the endotoxin levels for single and multiple-session using calcium hydroxide medication irrespective period of time of application.

**Figure 5:**
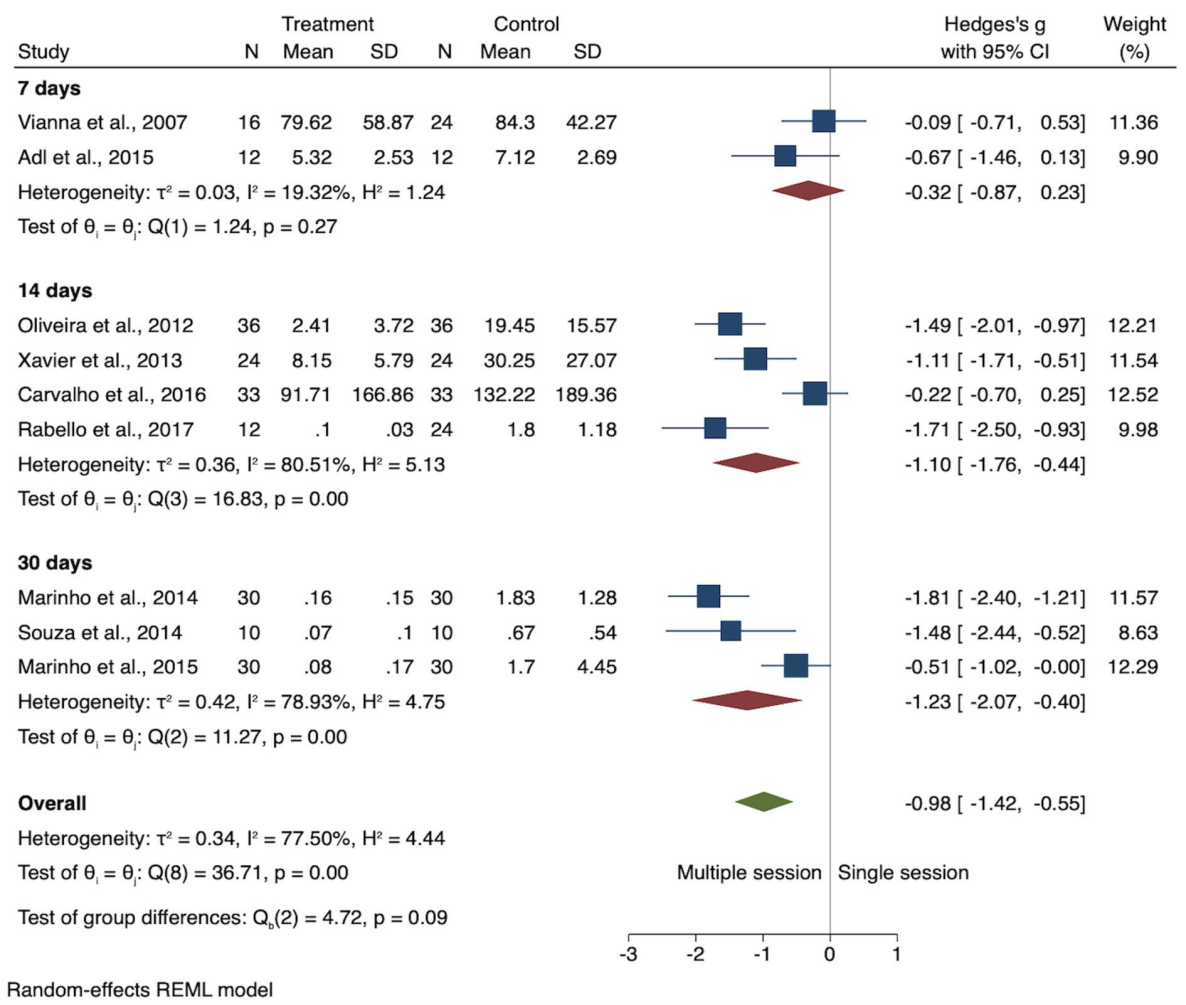
Forest plot for the endotoxin levels for single and multiple-session using calcium hydroxide medication for 7, 14, and 30 days.

Among the 9 studies included in this SR, four studies provided data in median values^1,12,22,36^. Such authors were contacted to obtain raw data for the calculation of the mean values. Seven out of 9 studies reported significantly higher levels of endotoxin reduction achieved with the placement of Ca(OH)_2_ intracanal medications when compared to single-session irrespective period of time of application^10,12,18,20,22,36,37^. Whereas, Vianna 2007^25^ and Carvalho 2016^21^ showed no significant difference in the reduction of endotoxin after the placement of Ca(OH)_2_ intracanal medication for 7 days and 14 days, respectively. The authors indicated an increase in the reduction of endotoxin levels in 14%^25^ and 25.6%^21^, respectively. Figure 4 shows the forest plot for the endotoxin levels for single- and multiple-session treatments irrespective the period of Ca(OH)_2_ intracanal medication application. Despite the heterogeneity across the 9 studies (I^2^ = 77.5%; *P* < 0.001), the overall meta-analysis indicated lower levels of endotoxin achieved with multiple-session than in single-session treatment (SMD − 0.98; *P* < 0.001) (Fig. 4). The sub-group analysis indicated no difference between single-session and 7 days of Ca(OH)_2_ medications (SMD − 0.32; *P* = 0.22). However, 14-days (I^2^ = 80.5%, *P* < 0.001) and 30-days (I^2^ = 78.9%, *P* < 0.01) of Ca(OH)_2_ medications was more effective than single-session treatments (both, *P* < 0.001). Figure 5 shows the forest plot for the endotoxin levels found in single and multiple-session treatment (7, 14, and 30 days) as well as the overall estimated effect for all the period of times combined.

## Discussion

This SR addressed the following common clinical question—What is more effective in reducing or eliminating endotoxin in endodontic infections—single or multiple-session treatments using calcium hydroxide medications? For that, we combined clinical studies that used calcium hydroxide intracanal medications for 7, 14, and 30 days over single-session protocols to get a broad answer about their effectiveness against endotoxin present in root canal infections. Additionally, 7, 14, and 30 days subgroup meta-analysis were performed to estimate the effectiveness of Ca(OH)_2_ intracanal medication for each period of time.

Keeping participants, and healthcare providers delivering the intervention unaware of the assigned intervention—a process known as blinding or masking—can reduce the risk of bias due to deviations from the intended intervention. Depending on the type of outcome measurement selected, the blinding can influence outcomes, in particular those studies that participant reports outcomes, observer makes judgment and/or provider makes decision on outcomes. In this SR, we looked at the difference in the endotoxin levels after single and multiple-session protocols with the placement of Ca(OH)_2_ intracanal medications, which is considered observer-reported outcomes with no judgement involved.

Here, the overall estimated effect for all the data combined from the 9 studies indicated that when Ca(OH)_2_ intracanal medications was used in multiple-session treatment protocols, endotoxins levels were lower than those obtained after single-session treatment protocols irrespective the period of time of application. The pool estimated effect for all the data combined, indicated by the ‘diamond shape’ in the forest plot, fell in the left side on Fig. 4, which here means in favor of the multiple-session protocols with the placement of Ca(OH)_2_ intracanal medications over single-session treatment protocols as labeled. The overall meta-analysis (Fig. 4) indicated considerable heterogeneity. Such heterogeneity was expected, and for that reason, we pre-planned subgroup meta-analysis for each period of time (7, 14, and 30 days), considering that the time of application could be an important modifier effect.

It is important to highlight that although only 2 studies were included in the 7-day subgroup meta-analysis, both studies are very consistent, indicating low heterogeneity between them (I^2^ = 20.4%; Chi^2^ = 0.35, *P* = 0.555). Both single studies indicated an increase in the reduction of endotoxin in 14%^25^ and 25.6%^20^, respectively. In contrast, considerable heterogeneity was detected among the studies for both 14 days (I^2^ = 82.2%; Tau^2^ = 0.4102; Chi^2^ = 16.76, *P* = 0.001) and 30 days (I^2^ = 82.3%; Tau^2^ = 0.5399; Chi^2^ = 11.24, *P* = 0.004) of Ca(OH)_2_ medication protocols. According to the I^2^ results, the heterogeneity for both 14 (82.2%) and 30 (82.35%) days is slightly higher than the overall combined data level at 78.1%. Therefore, in particular, to 14 and 30 days, the difference in time of application has not explained the observed heterogeneity.

Since this SR was designed to address a clinical question, two main types of heterogeneity rise (1) clinical heterogeneity, in which differences between the studies may relate to interventions, and (2) methodological heterogeneity that indicates differences in the type of study design. The clinical heterogeneity (1) among the14 and 30 days studies may be attributed mainly to the intracanal medication, including the type of vehicle, the medication dosage (ratio of preparation), the renewal of the medication, as well as the method of delivery. The type of vehicle for the medication used across the studies was either 2% CHX-gel^18,21,25,36^, saline solution^10,12,20,25,37^ or propylene glycol^22^. Although 2% CHX-gel has no detoxifying effect against endotoxin^38^, its viscous vehicle, in a gel formula, influence the dissociation of Ca^+2^ and Cl^–^ ions. The medication dosage, the ratio of Ca(OH)_2_ to the vehicle, was 1:1^10,18,21,37^ or not described^12,20,22,25,36^. Different study designs (e.g., RCT versus NRSis) might produce different results, which might result in methodological heterogeneity (2). However, such heterogeneity is limited in this SR, once all studies included were classified as ‘low’ risk of bias, and therefore with a low possibility that the effect size is overestimated^39^.

All 9 studies included here used the Limulus amebocyte lysate (LAL) method to quantify the levels of endotoxin in root canal infection. The LAL method uses a serine protease catalytic coagulation cascade that is activated by endotoxin. Endotoxin binds to the first component in this cascade, Factor C, which is a protease zymogen. Subsequently, the cascade induces the activation of a proclotting enzyme (coagulaten) into a clotting enzyme (coagulin). Such a clotting enzyme cleaves the synthetic peptide-pNA substrate, used in the chromogenic LAL assays (QCL or KQCL), and imparts a yellowish color to the solution. As coagulogen converts into coagulin, a gel clot begins to be formed, and therefore, increase the turbidity, which is captured by the turbidimetric kinetic assay. Then, the concentration of endotoxin is determined by the strength of the yellow color (determined at an OD = 340 nm) resulting from the chromogenic LAL and the turbidity (determined at an OD = 340 nm) resulting from the coagulogen conversion. These three tests differ in the technique to detect endotoxin. The two chromogenic tests (QCL-1000 and KQCL tests) use synthetic peptide-pNA substrate, which is cleaved by the clotting enzyme, making the solution yellowish in color. While the turbidimetric test (Pyrogent 5000 test) monitors, the increase of the turbidity resulted from the conversion of coagulogen in coagulin. The two Chromogenic LAL tests determine the concentration of endotoxin in an optical density (OD) at 405 nm and while the turbidimetric LAL test in an OD at 340 nm. Both the Kinetic methods (KQCL test and Pyrogent 5000 test) monitors the progress of the LAL reaction, which confers a wider sensitivity range (0.01–100 EU/mL^−1^). While for the Endpoint method (QCL-1000 test), the quantification of endotoxin is determined at a time point, specifically at 16 min after the initiation of the LAL reaction, which limits its sensitivity (0.1–1 EU/mL^−1^). The variations of the type of endotoxin test selection across the studies included in this SR might have influenced on the effect size in terms of endotoxin quantification. According to Martinho 2011^40^, the KQCL test yielded a median value of endotoxin close and not significantly different from the turbidimetric test. However, the endpoint QCL test indicated ~ 5 × greater than the KQCL and turbidimetric test^40^.

Previous studies investigated the ability of different irrigants to reduce endotoxin levels in root canal infections^41,42^. Buck 2001^41^ compared in vitro the detoxifying activity of most commonly used irrigants against lipid A present in endotoxin, and the authors found little or no detoxifying activity. In agreement, Martinho 2008^42^, in a clinical study, revealed no difference between NaOCl and CHX-gel in the elimination of endotoxin from infected root canals. The authors attributed the removal of endotoxins from dentin walls to the mechanical action of files against the dentin walls and the irrigant's flow and backflow inside the canal rather than the irrigant's detoxifying activity. Moreover, Herrera 2017^43^ showed that the ultrasonic activation of EDTA was effective in further reducing endotoxin levels in the root canals of teeth with pulp necrosis and apical periodontitis.

One of the limitations of this SR is the lack of information in few studies about the sample size calculation as well as the type of randomization, which could possibly result in a definitive judgment of “moderate” or “high” risk of bias. However, the authors were contacted to obtain the missing information to enable a definitive judgment of ‘low’ bias. Although few studies included here lack of information regarding sample size calculation, a minimum of ten patients per group was reported in all studies except for Vianna 2007^25^. Ten patients per group seem to be enough samples to avoid them from being underpowered.

## Conclusion

Overall, this SR provides evidence to support that multiple-session disinfection protocols with the placement of Ca(OH)_2_ intracanal medications are more effective in reducing the levels of endotoxin from root canal infections compared to single-session when applied for 14 and 30 days. At present, there is no substantial evidence of the advantage of the 7 days Ca(OH) intracanal medication protocol over single-session treatment.

## Supplementary Information

Supplementary Table S1.
