# Supplementary material for: Comparison of the effectiveness of single- and multiple-sessions disinfection protocols against endotoxins in root canal infections: systematic review and meta-analysis
Source: Sci Rep. 2021 Jan 13;11:1226. doi: 10.1038/s41598-020-79300-3 (PMC7806761; doi:10.1038/s41598-020-79300-3)
Supplement: Supplementary file 1 — Supplementary Table S1. [file 41598_2020_79300_MOESM1_ESM.doc]

Table S1 - Excluded studies and reason for exclusion
